# Chemical, Thermo-Mechanical and Antimicrobial Properties of DBD Plasma Treated Disinfectant-Impregnated Wipes during Storage

**DOI:** 10.3390/polym11111769

**Published:** 2019-10-27

**Authors:** Xinyu Song, Uros Cvelbar, Petra Strazar, Lutz Vossebein, Andrea Zille

**Affiliations:** 1 2C2T-Centro de Ciencia e Tecnologia Textil, Universidade do Minho, Campus de Azúrem, 4800-058 Guimaraes, Portugal; xinyu.song@gmx.de; 2 Jozef Stefan Institute, Jamova cesta 39, SI-1000 Ljubljana, Slovenia; uros.cvelbar@guest.arnes.si (U.C.); petra.strazar@gmail.com (P.S.); 3 Faculty of Textile and Clothing Technology, Niederrhein University of Applied Sciences, Webschulstrasse 31, 41065 Mönchengladbach, Germany; lutz.vossebein@hs-niederrhein.de

**Keywords:** dielectric barrier discharge (DBD), pre-wetted disinfecting wipes, ageing, ready-to-use disinfectant wipe, antimicrobial, quaternary ammonium compounds, plasma

## Abstract

Disinfectant-impregnated wipes are broadly used in hospitals, as an important approach for infection prevention and control. But their ageing performance has rarely been studied. Untreated and Dielectric Barrier Discharge (DBD) plasma pre-treated wiping materials made of nonwoven 100% polyester (W1), nonwoven 55% cellulose/45% polyester (W2) and woven cotton (W3) were impregnated with a quaternary ammonium compound solution (ADBAC) for 30 min, 3, 7, 15, and 30 days of storage time and characterized in term of chemical, thermo-mechanical and antimicrobial efficacy. X-ray photoelectron spectroscopy analysis on the plasma-treated polyester wipes demonstrates the incorporation of reactive oxygen species on the fiber surface. Laser scanning microscopy demonstrates the plasma etching effect in smoothing the surface of the cotton wipe reducing the adsorption of ADBAC. The result showed no significant changes in breaking force and elongation during storage for W1 and W2. However, plasma treatment affects W3 in weft direction reducing the force at break in water and ADBAC treated wipes. Dynamic mechanical analysis results show that ADBAC and plasma treatment have a significant influence in W1 and W3 viscoelastic properties improving the elastic response limiting the polymeric chains mobility and the non-elastic response due to the etching effect, respectively. Overall, the plasma pre-treatment of ADBAC-impregnated wipes is able to enhance the antimicrobial performance and the storage time of polyester-containing wipes.

## 1. Introduction

Surface disinfectants integrated with textile materials as disinfectant-impregnated wipes are the most prevalent disinfection methods used in nosocomial environment, food processing industry and other domestic situations [1,2,3]. Their simple application, reliable performance and user-friendly features contribute to their wide-spreading for surface disinfection application [2]. Particularly, the participation of textile material could remove visible debris and organic matters that could hinder the disinfectant performance [3,4]. Typical disinfectants found in the market are alcohol, chlorine, and chlorine compounds (hypochlorite, chlorine dioxide, and chloramine-T), hydrogen peroxide, peracetic acid, and quaternary ammonium compounds (quats or QACs) [4,5]. The most commonly used textile material in wipe application is composed by cellulosic fibers such as cotton, woodpulp, viscose etc., and thermoplastic fibers like polyester and polypropylene [6,7].

Adsorption of disinfectants due to the presence of certain textile materials have been previously reported in the literature [8,9,10]. The use of an inappropriate wipe material (e.g., cellulosic material) could interact with the absorbed active ingredient (e.g., quats) resulting in lowering, or even inhibiting, the disinfectant efficacy [9]. In spite of this, some research has been performed on the effectiveness of commercially available disinfecting wipes in clinical use, it remains unknown regarding the ageing of disinfectant-impregnated wipes in storage [11,12]. In polymer material, five types of ageing classifications exist, physical ageing, photochemical degradation, thermal degradation, chemical attack, and mechanical stress [13]. For professional use (industry, hospital, healthcare centre, etc.), the disinfectant-impregnated wipes have to be stored in a shaded, cool, dry, stable and well-ventilated place. Thus, photochemical, thermal and mechanical degradations usually have a low impact and physical ageing occurs all along the lifetime of the product [13,14]. However, to the best knowledge of the authors, the understandings of the storage chemical ageing of wipes in the presence of disinfectant are very poor. Chemical attack on textiles due to disinfectants can change the material properties from soft to hard and stiff or lowering the lint levels [13]. Furthermore, the interaction between wipe material and disinfectant during storage can also affect the products’ disinfection performance. The following questions remain to be answered: (i) How does the wiping materials degrade in terms of structure, properties and function? (ii) How does the adsorption of active ingredients onto textile substrate change with storage time? and (iii) How does the antimicrobial efficacy vary by time?

Plasma treatment has been extensively applied for surface modification of textile material. It is a dry, environmentally- and worker-friendly method to achieve surface alteration without modifies the bulk properties of different materials [15,16]. In the last years, atmospheric cold plasma, as dielectric barrier discharge (DBD), was widely employed because it does not need expensive vacuum equipment and allow continuous and uniform processing of fibers surfaces [17]. The DBD plasma treatment has been reported in the literature improving the surface energy and surface oxidation of polyester material [18]. For cellulose material, DBD plasma treatment usually provides only an etching effect in terms of surface ablation and surface morphology change [19]. However, it was recently reported that DBD plasma not only effectively cleaning cotton fibers, increasing roughness and wettability but also increase the polar surface functional groups and alter the surface charge [20,21]. All these possible changes resulting from plasma treatment could have an impact on the wipe material properties and the adsorption of quats onto textile materials. Hence, DBD plasma treatment was considered as a possible solution to reduce the binding of quats and to improve absorption onto wipe materials.

This paper studied the ageing of the untreated and DBD plasma-treated disinfectant-containing wipes after 30 min, 3 days, 7 days, 15 days and 30 days of storage. Quaternary ammonium compounds (ADBAC) as the disinfectant and 3 commercial wiping materials of polyester (PET), 55% cellulose/45% PET (CELPET) and 100% cotton (CO) have been selected. X-ray photoelectron spectroscopy (XPS) was used to assess the chemical interaction between ADBAC and untreated and plasma-treated wipe samples. The concentration reduction of bulk ADBAC solution before and after wipe immersion at different aged time was analyzed by the means of UV-Vis spectrophotometry. Fourier-transform infrared spectroscopy (FTIR) and Dynamic mechanical analysis (DMA) were used to evaluate the interaction of ADBAC with wipe materials for a certain storage time. Breaking force and elongation change were also recorded with a universal testing machine (UTM). Finally, the antimicrobial efficacy changes of disinfectant impregnated wipes along storage time was investigated based on the test standard ASTM E 2149-13a [22]. By studying the ageing performance of the disinfectant-impregnated wipes it was noted that plasma treatment can increase the ADBAC concentration on the polyester wipe surface. Moreover, it was also possible to prolong the antimicrobial efficacy of polyester-containing wipe samples during storage time by increasing the adsorption of ADBAC and slowly releasing the adsorbed ADBAC over time.

## 2. Materials and Methods

### 2.1. Materials

The quaternary ammonium salt used for the tests is alkyldimethylbenzylammonium chloride (ADBAC) and it was purchased from the company EMD Millipore Corporation, a subsidiary of Merck KGaA, Germany and reserved in a plastic bottle in solid status. The chemical formula is C_6_H_5_CH_2_N(CH_3_)_2_RCl (where R = C_8_H_17_ to C_18_H_37_). Once the bottle was open, it was reserved in the desiccator containing silica gel desiccants to avoid the degradation from humidity. The commercial wipe samples (W1 and W2 in Table 1) used in this project belong to the category of disposable and semi-disposable wiping cloths (areal density of below 80 g m^−2^). W1 is TX409 Absorbond^®^ (Texwipe Inc., Kernersville, NC, USA) and W2 is Wipe EcoCloth (Contec Inc., Spartanburg, SC, USA). White bleached cotton woven fabric with a warp density of 34 threads cm^−1^, a weft density of 30 threads cm^−1^ was used. All other chemicals were purchased from Sigma-Aldrich (Waltham, MA, USA) and used without further purification steps.

### 2.2. Sample Characterization

Wipes were pre-washed with 0.05% non-ionic detergent Diadavin UN (Tanatex chemicals, Ede, The Netherlands) in a standard washing process carried out by a long bath equipment, model IBELUS IL-720 (Labelus, Braga, Portugal) integrated with an infrared heating system. The program started at 20 °C and rose up to 40 °C with a gradient of 3 °C min^−1^, and the temperature remained for 30 min. The liquor ratio for the washing process is 1:100 (fabric mass in g: detergent solution in mL) with an agitation speed of 40 rpm. Then, the samples were rinsed with distilled water by 1:100 liquor ratio three times. Afterwards, wipe samples were placed in an oven at 40 °C for 24h to dry for further use. Fabric thickness and area density were determined at the standard atmosphere of 20 ± 2°C and 65± 2% RH. All wipe samples were conditioned for 48 h before testing. The thickness of the fabrics was measured according to the standard ASTM D1777-96 (2015) with the digital thickness gauge M034 A at a pressure of 100 Pascal [23]. Every sample was repeated 10 times and the mean and coefficient of variance were calculated. A circular cutter with a surface area of 100 cm^2^ was used to prepare the sample wipes for further areal density measurements. Every sample wipe was taken 5 times measurement. The data are reported as mean ± coefficient of variance% (CV).

### 2.3. DBD Plasma Treatment of Wipe Samples

**Figure A1:**
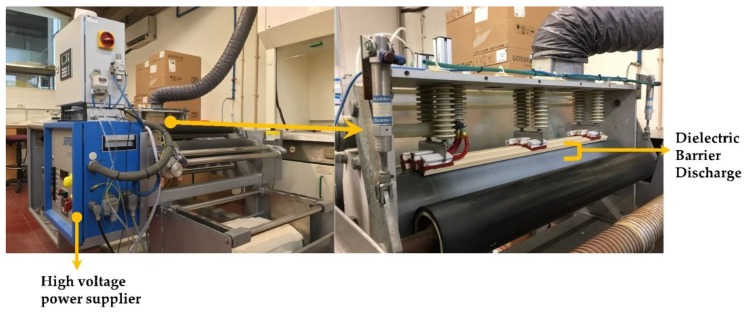
Schematic diagram with a photo of the DBD plasma equipment used for the sample treatment.

The dielectric barrier discharge (DBD) plasma treatment was conducted in a semi-industrial prototype (Softal Electronics GmbH/University of Minho, Braga, Portugal) working at room temperature and atmospheric pressure, using a system of metal electrode coated with ceramic and counter electrodes coated with silicon, with 50 cm effective width, gap distance fixed at 3 mm, and producing the discharge at high voltage 10 kV and low frequency 40 kHz. The machine was operated with the optimized parameters: 1 kW power, 4 m min^−1^ velocity, 5 passages corresponding to a dosage of 2.5 kW min m^−2^ adopted from the previous study [24]. Plasmatic dosage was defined by Equation (1):

(1) $$\mathrm{Dosage}=\frac{N\cdot P}{v\cdot l}$$

where, N = number of passages, P = power (W), v = velocity (m min^−1^), and l = width of treatment (0.5 m). The wipe was passed through a laminar plasma between a cylindrical silicone rotating drum and ceramic electrodes for one side then treated on the other side. A schematic diagram using a photo of the used equipment was provided in Figure A1 in the Appendix A.

### 2.4. Contact Angle Measurement

The contact angle measurement of the wipe samples before and after dielectric barrier discharge (DBD) plasma treatment was performed with an OCA apparatus from Dataphysics Instruments GmbH (Filderstadt, Germany), associated with the OCA20 software. Measurements were done with 10 replicated on every sample and the average and standard deviation were calculated.

### 2.5. Laser Scanning Microscope (LSM)

LSM was performed on cotton woven wipe to characterize the surface roughness before and after plasma treatment. An LSM from Keyence microscope (Osaka, Japan), model VK-X160 with a red semiconductor laser supplied at wavelength 658 nm was used. The surface roughness was evaluated from the measurement of arithmetical mean height (Sa) and developed interfacial area ratio (Sdr). Scanning was stitched 80 times and an area of 10 mm^2^ of the untreated and plasma-treated wipe samples were analyzed.

### 2.6. Storage of Wipe Samples in ADBAC Solution

ADBAC solution was prepared freshly at the concentration of 0.8 g L^−1^ by adding 0.8 g (±0.5%) of ADBAC to a 1000 mL volumetric flask filled up with distilled water. The wipe samples were immersed in the prepared ADBAC solution in the liquor ratio of 1:20 (fabric mass g: bulk solution mL). To fulfil the sample requirement from UTM measurement, two pieces of wipe sample with 0.5gram mass were immersed in 40 mL ADBAC solution. Wipe samples and ADBAC solution was maintained in a 50 mL conical tubes and stored in a shaded, cool, dry, and well-ventilated cabinet for 30 min, 1 day, 3 days, 7 days and 30 days. When the due time elapsed, wipe samples were taken out by a tweezer and dry in the oven at 40 °C for 24 h for further tests. The remained bulk solution was analyzed by UV-Vis spectrophotometry. For the ASTM E 2149-13a test, wipes were cut in samples of 0.05 g (Table 1) [22]. Six pieces of the same wipe samples were immersed in 6 mL ADBAC solution in a 15 mL volume high-density polypropylene conical tubes with an adjustable pipette (1 to 10 mL). Every wipe sample set was coupled with water control under the same experimental conditions.

### 2.7. X-Ray Photoelectron Spectroscopy (XPS)

The XPS analyses were carried out on the PHI-TFA XPS spectrometer produced by Physical Electronics Inc., Chanhassen, EUA. Wipe samples with immersion time 30 min (D0) were selected for evaluate the chemical interaction between the ADBAC and untreated and plasma- treated wipe samples. Samples were mounted on the metallic sample holder and introduced in ultra-high vacuum spectrometer. The vacuum during the XPS analyses was in the range of 10^−9^ mbar. The analysed area was 0.4 mm in diameter and the analysed depth was about 3–5 nm. All the measures were taken with an angle tilt of 45°. Sample surfaces were excited by X-ray radiation from monochromatic Al source at photon energy of 1486.6 eV. The survey wide-energy spectra were taken over an energy range of 0–1400 eV with a pass energy of analyser of 187 eV in order to identify and quantify present elements on the surface. Two places on every sample were analysed and average composition was calculated. The smallest concentration of elements which can be detected with XPS method (sensitivity) is about 0.5 at.%. XPS spectra were analysed for elemental composition by a Multipak software, version 8.0 from Physical Electronics Inc. company, Chanhassen, EUA.

### 2.8. Spectrophotometric Assessment of ADBAC Concentration

The concentration of ADBAC was assessed by UV-Vis spectrophotometry (Shimadzu, Kyoto, Japan, UV-1800) with quartz cuvettes. Initially, a full wavelength scans from 200–800 nm was performed to determine the λ_max_. The ADBAC solution showed three peaks at 268.5, 262.5, and 256.5 nm. The wavelength at 262 nm was chosen for its highest response. A calibration curve of concentration [C] versus absorbance (abs) was developed. The obtained calibration equation was [C] = 0.806 Abs − 0.007 (R^2^ = 0.9999). This formula was adopted to calculate the concentration of ADBAC solution in the subsequent tests. The UV-Abs of every ADBAC solution after wipe removal at all aged time were recorded.

### 2.9. Fourier Transform Infrared Spectroscopy (FTIR)

The FTIR spectra of untreated/plasma-treated wipes samples immersed in water/ADBAC at all aged time were recorded using an IR-Affinity 1 FTIR spectrophotometer (Shimadzu, Kyoto, Japan) with an attenuated total reflectance accessory (ATR) to determine the surface chemical changes. Spectra were collected at room temperature in the spectral range of 4000–700 cm^−1^ at the resolution of 4 cm^−1^ and summations over 45 scans. All the samples were dried in an oven at 40°C for 24h prior testing.

### 2.10. Breaking Force and Elongation Measurement

Fabric strain versus applied force and time was measured (at 20 °C and 65% RH) with a Universal Testing Machine (Model 4500, Instron Corporation, Norwood, MA, USA) using a 250 N load cell at the crosshead speed of 1 mm min^−1^. Samples of 2 × 10 cm were tested in warp and weft (or cross and machine) directions at the maximum load of 250 N using the standard test method for breaking force and elongation of textile fabrics (Strip Method, ASTM D 5035:11 2019) [25]. Wipe samples at all aged time were tested.

### 2.11. Dynamic Mechanical Analysis (DMA)

DMA was conducted with a DMA 7100 from Hitachi^®^ (Tokyo, Japan) in programmed tension mode. The storage and loss moduli, tan delta was measured in the temperature range 30 to 200 °C, with a heating rate of 3 °C min^−1^. The geometry of the testing sample was set 20 mm in length, 10 mm in width and the thickness in mm of each wipe as reported in Table 1. Specimens were prepared in duplicate to conduct thermo-mechanical analysis. These analyses were carried out under nitrogen purge of 200 mL min^−1^. 7 days’ ADBAC/water immersed untreated and plasma-treated wipe samples were investigated by DMA. The wipe samples were dried in an oven at 40 °C for 24 h prior testing.

### 2.12. Microbiology Test ASTM E 2149-13a

The ASTM E 2149-13a Standard Test Method for Determining the Antimicrobial Activity of Immobilized Antimicrobial Agents under Dynamic Contact Conditions was modified to assess the antimicrobial efficacy change over storage time [22]. The most important modification of the standard was the enhancing of the bacteria inoculum concentration in order to allow more evident distinction in differentiating the antimicrobial performance among the three wipe samples. Therefore, the bacteria inoculum concentration was rose up between 1.5 to 3.0 × 10^9^ CFU mL^−1^. However, other modifications with the intent of saving material due to the high number of used samples and bacterial suspensions were made in terms of wipe sample size and volume of bacteria inoculum. The wipe sample size was defined at 0.05 g and the volume of the bacteria inoculum was reduced to 5 mL. Due to the different areal densities of wipe sample materials, the approximate dimension of wipe samples is reported in Table 1.

The working bacteria are *Staphylococcus aureus* (*S. aureus*), ATCC 6538 and *Escherichia coli* (*E. coli*), ATCC 25923 representing Gram-positive and Gram-negative bacteria, respectively. The stock culture was prepared from the freeze-dried ampule into several vials and stored at −80 °C. A subculture (G1) was prepared from the frozen stock culture on tryptone soya agar (TSA) plates and incubated at 37 °C for 24 h. The working culture for the test is freshly prepared from G1 in the sterile Tryptic Soy Broth (TSB) for 18 h at 35 ± 2 °C prior to performing the test. The testing samples were prepared in three repetitions, obtained from the wipes immersed in ADBAC solution under storage conditions (at 30 min, 3, 7, 15, 30 days). The testing samples with 5 mL bacterial suspension were maintained in a 15 mL sterilized conical tubes for 1 h with orbital shaking at 120 rpm and 37 °C. Afterwards, liquids were collected from each tube for plating. Plates were incubated at 35 ± 2 °C for 18–20 h. The plate counting, log reduction calculation and other detailed test procedure were performed according to the standards.

## 3. Results and Discussion

### 3.1. Chemical Interaction Assessment (XPS Analysis)

**Figure A2:**
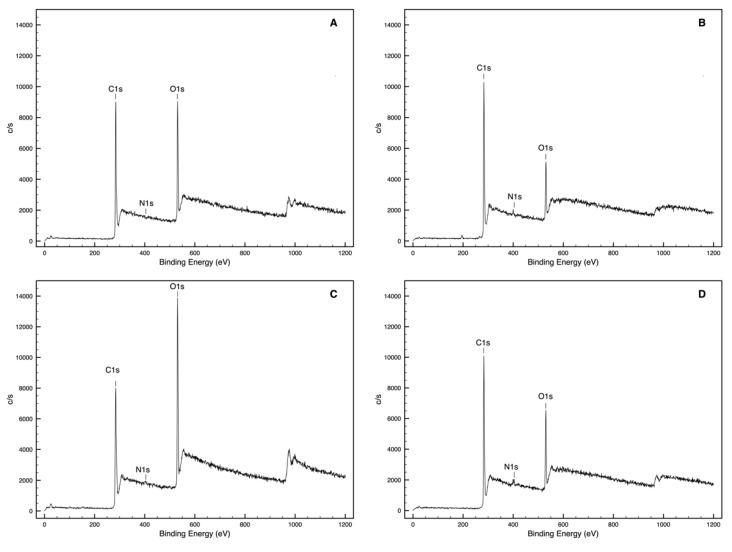
XPS survey scan of polyester nonwoven wipe (W1), (**A**) W1R, (**B**) W1RQ, (**C**) W1P, (**D**) W1PQ.

**Figure A3:**
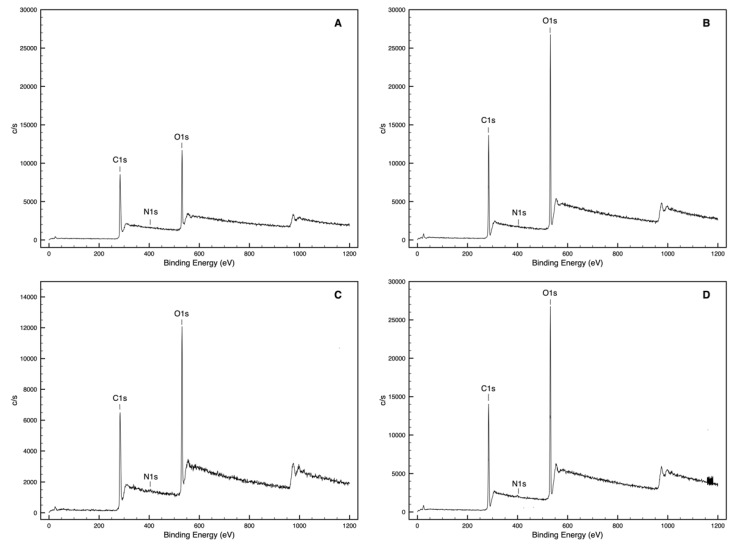
XPS survey scan of cellulose/polyester nonwoven wipe (W2), (**A**) W2R, (**B**) W2RQ, (**C**) W2P, (**D**) W2PQ.

**Figure A4:**
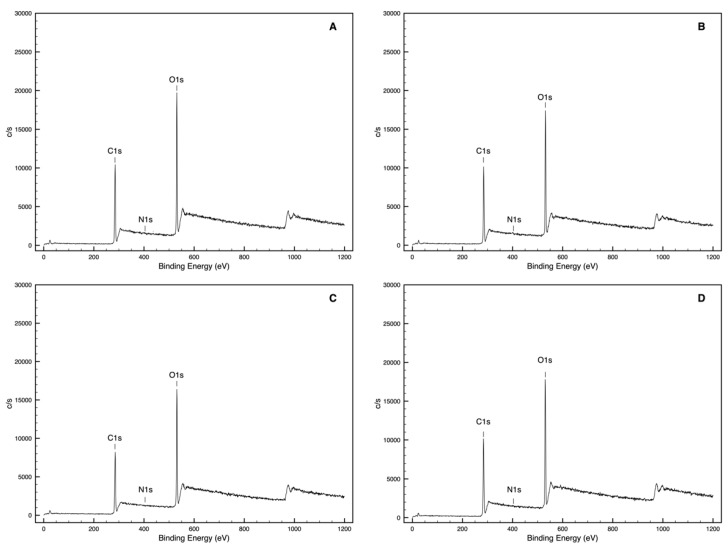
XPS survey scan of woven cotton wipe (W3), (**A**) W3R, (**B**) W3RQ, (**C**) W3P, (**D**) W3PQ.

The degree of chemical modifications on the surface of the wipes was studied by XPS (Table 2 and Figure A2, Figure A3 and Figure A4 in the Appendix A). The relative chemical composition (C, N, O) and oxygen and nitrogen atomic ratios (O/C and N/C) were exhibited in Table 2. Plasma-treated wipes containing polyester (W1 and W2) were significantly altered in terms of oxygen content showing an increase of O/C ratio about 42% and 18% for W1 and W2, respectively. DBD plasma discharge in air at atmospheric pressure is able to generate a wide range of active species such as atomic oxygen, ozone, nitrogen oxides and radicals. After plasma treatment, the considerable increase in the oxygen content is due to the incorporation of oxygen-containing polar groups onto the polyester fibres surface generating hydroxyl and carboxyl groups [26,27]. W3 shows only a slight increase of about 5% in O/C ratio confirming that this DBD plasma is not able to substantially oxidize the cellulose polymer chain. Most of the work demonstrating cotton surface oxidation by atmospheric plasma in the air were developed using raw cotton that contains several non-cellulosic components in cuticle and primary wall [20,28]. However, in this work, a white bleached woven cotton was used preventing further surface oxidation. Some nitrogen was detected in the untreated wipes control surfaces probably due to contamination. After plasma treatment, there is a small increase in nitrogen relative atomic percentage resulting from the introduction of air nitrogen from the atmosphere, as previously reported [29]. However, all the wipes did not show any significant difference in the N/C ratio.

**Figure A5:**
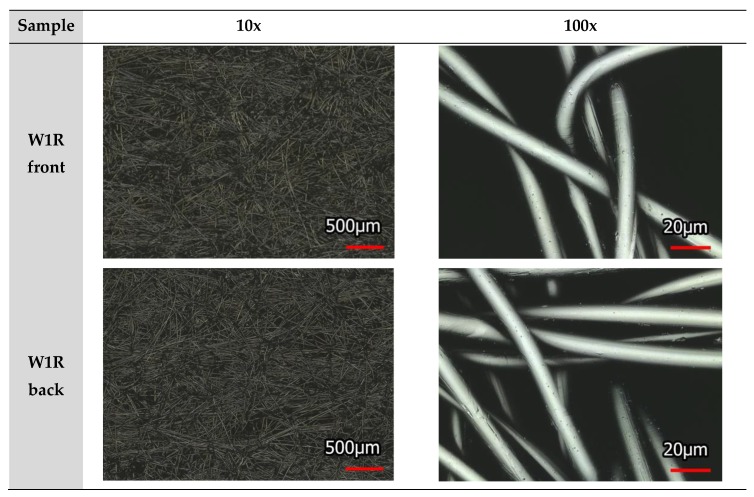

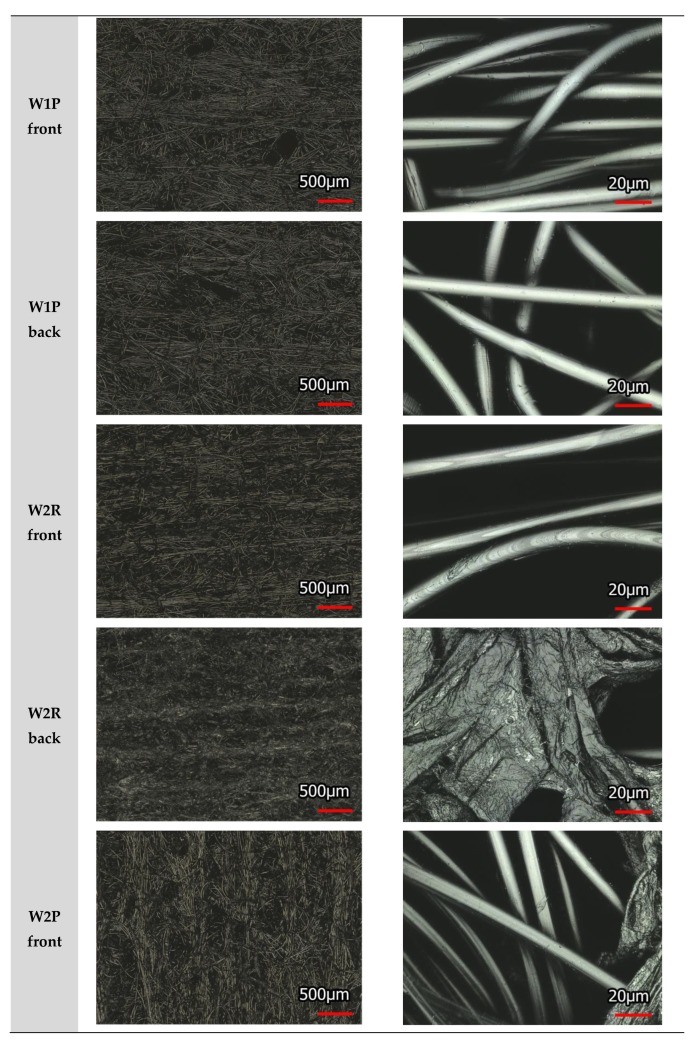

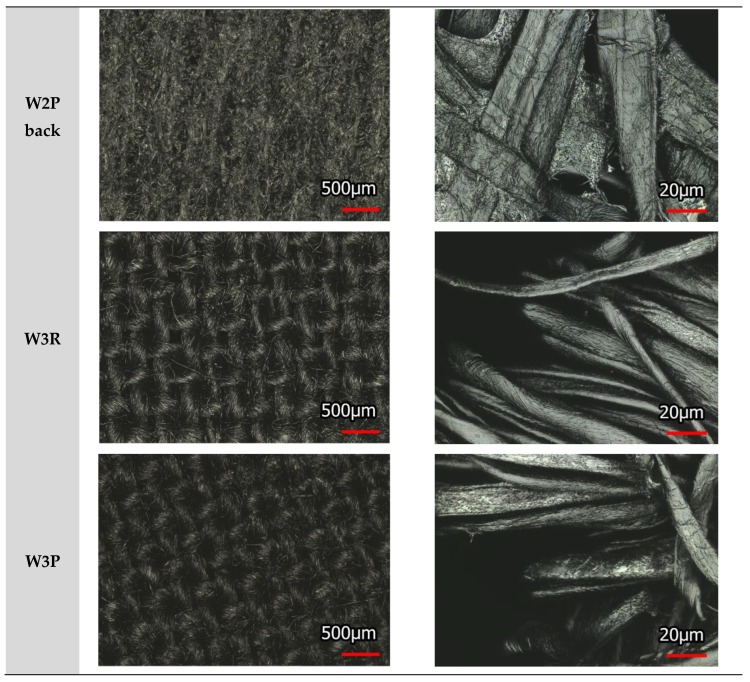
Laser Scanning Microscopic Images of the untreated and plasma-treated wipes in both sides (only for nonwovens) at magnifications of 10× and 100×.

After the introduction of ADBAC on the wipes, the nitrogen content increased, as expected since it is part of the quaternary ammonium salt molecule. Polyester-based W1 wipe (W1Q) showed the higher amount of nitrogen content (1.7%). This is due to the higher hydrophobicity of pure polyester compared to the other wipe materials which hamper ADBAC solution to be absorbed in the bulk of the wipe resulting in higher ADBAC concentration on the wipe surface. After plasma treatment the nitrogen content increase of about 30% due to the plasma-induced oxygen species present onto the polyester fibres surfaces that can both improve wipe wettability and also adsorption of the ADBAC molecules. The cellulose/polyester blend W2 and the cotton W3 wipes showed lower nitrogen content compared to W1. Moreover, W2 and W3 nitrogen percentage decrease after plasma treatment suggesting a lower ADBAC adsorption due to the cleaning effect on the cellulose fibres by etching, removing the more hydrophilic surface microfibrils. This was confirmed by laser scanning microscopy (LSM) that shows, after plasma treatment, a decrease in both developed interfacial area ratio (from 6.74 to 4.93) and arithmetical mean height (from 31.49 to 25.91). LSM images of untreated and plasma-treated wipes at magnifications of 10× and 100× were presented in Figure A5 in the Appendix A.

### 3.2. Adsorption of ADBAC During Storage Time

**Figure 1:**
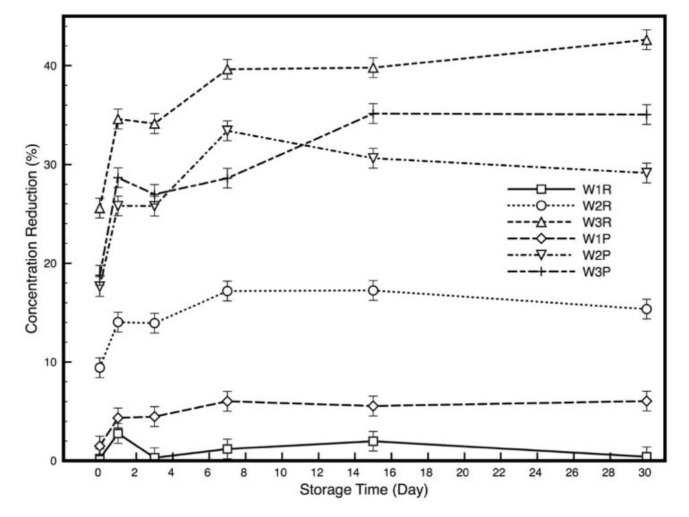
Concentration reduction in ADBAC bulk solution during storage time.

Figure 1 exhibits the concentration change of bulk ADBAC solution during storage time up to 30 days. For W1 (100% PET), both untreated and plasma-treated wipes showed extreme low adsorption (expressed as the percentage of concentration reduction in Figure 1). However, the plasma-treated wipe showed slightly higher adsorption than the untreated one. This can be explained by the plasma-induced increase of surface energy polar component by surface oxidation (as also observed by the fiber yellowing), which can enhance the interaction between polyester and ADBAC molecules [18]. Plasma treatment can generate active oxygen species on the polyester surface, such as hydroxyl groups, providing partially negative charge on the surface which can interact with the positively charged ADBAC molecules [30]. Meanwhile, the increased hydrophilicity of polyester samples improves the absorption of ADBAC solution on the textile substrate. The measured contact angle for W1 dropped from 147.6° to 0° after plasma treatment. All the other samples showed contact angle of 0° before and after plasma treatment. Due to these two reasons, which was previously confirmed by XPS analysis, the adsorption increased. Plasma treated polyester sample displays an evident increase in the adsorption up to 7 days, then it stabilized. Even though polyester samples showed an increase after plasma, its adsorption remains the lowest of the studied wipes.

W2 (CEL/PET) shows medium adsorption because of the negatively charged cellulose fibres interacting with the positively charged ADBAC active ingredient promoting adsorption. After plasma treatment, W2 wipes duplicated the adsorption ability (from 15% to about 30%) by the mutual action of the plasma oxidation of polyester fibres and by the already hydrophilic cellulose component. It seems that plasma treatment is able to enhance the cellulose absorption ability of W2 by increasing the liquid mass transfer into the inner part of the wipe. The swelling effect becomes more pronounced after plasma treatment facilitated by the shift of the cellulose shear plane towards the solution and furthermore increasing the adsorption [21,31].

W3 is a 100% cellulose wipe exhibiting the highest adsorption ability among all the wipe samples. However, plasma-treated W3 showed a decrease in adsorption compared to its control. On one hand, in literature, plasma treatment is efficiently used to clean raw cotton by removing its non-cellulosic components such as waxes, proteins and pectin, oxidizing its surface and increasing polar functional groups [20,32]. On the other hand, in this case, the used wipe was a white bleached cotton woven fabric that is already chemically oxidized as proved by XPS analysis (see Table 2). Plasma treatment reduces the surface roughness (see LSM results in Section 3.1) by etching the microfibrils on the cotton surface and promotes reorientation of the polar surface functional groups which significantly reduce the adsorption of ADBAC of around 10% [33].

Taking into account the XPS surface analysis results, it is noted that even though there is large adsorption of ADBAC in W2 and W3 (Figure 1), more ADBAC can be detected on the surface of W1 (Table 2), indicating that the absorbed ADBAC is more present on the surface of W1 comparing with the other two.

### 3.3. Chemical Change of the Wipe Surface Over Storage Time (FTIR)

**Figure 2:**
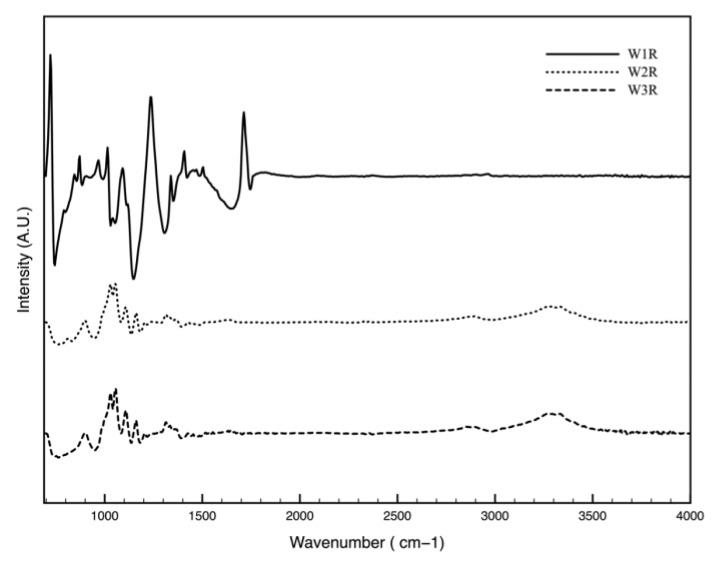
ATR-FTIR spectra (Intensity) of untreated W1, W2 and W3 samples in the spectral range between 700 and 4000 cm^−1^.

**Figure A6:**
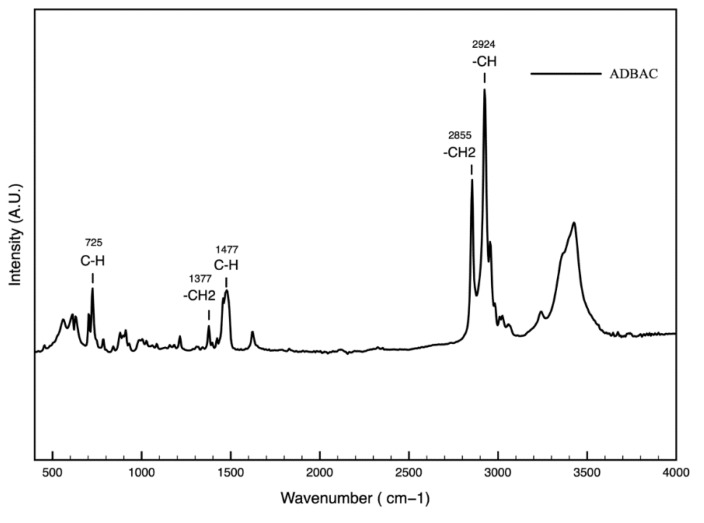
Diamond ATR-FTIR spectra of ADBAC in the range between 400 and 4000 cm^−1^.

**Figure A7:**
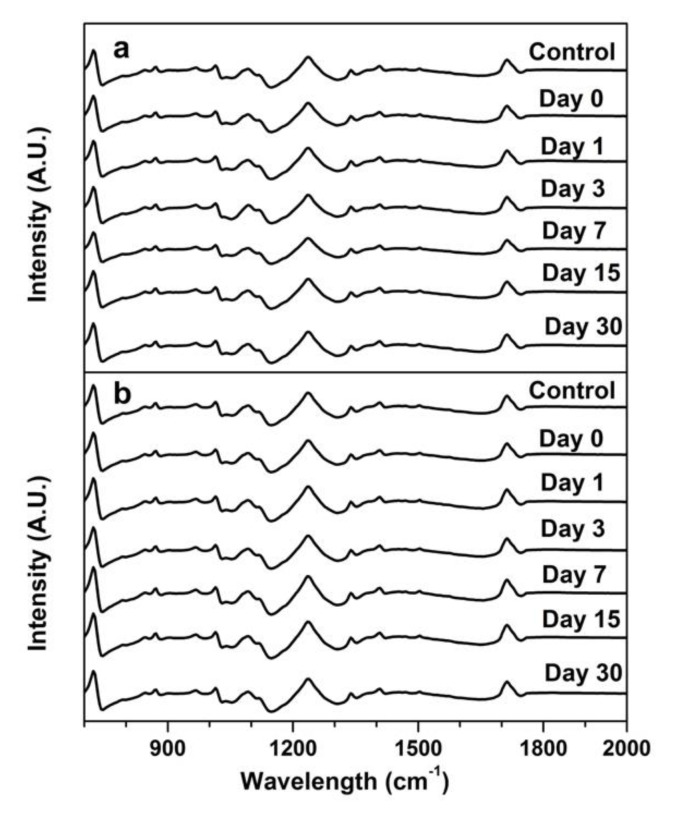
ATR-FTIR spectra of control (**a**) and plasma-treated (**b**) W1 (polyester) in the range between 700 and 2000 cm^−1^.

**Figure A8:**
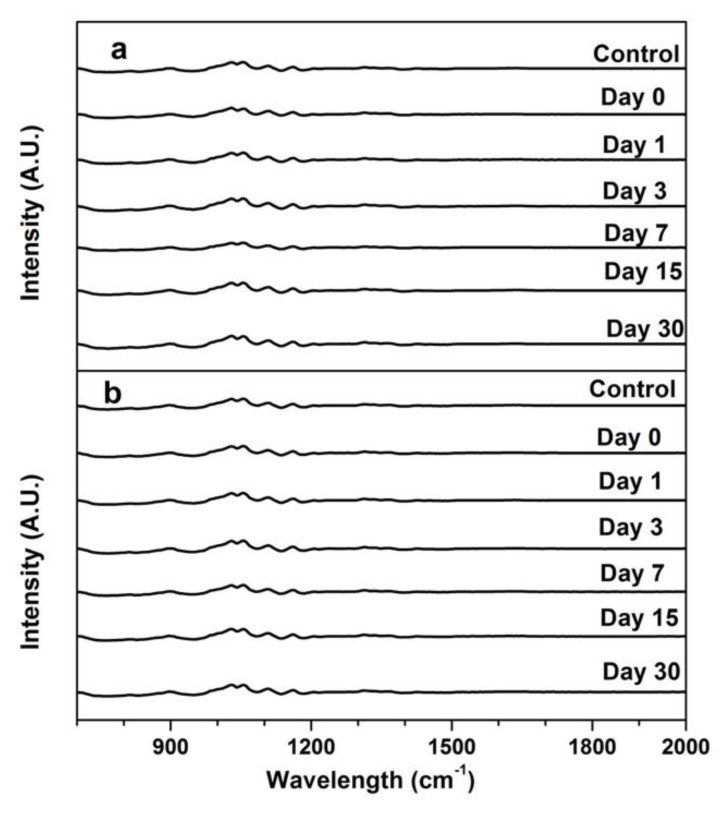
ATR-FTIR spectra of control (**a**) and plasma-treated (**b**) W2 (polyester/cotton) in the range between 700 and 2000 cm^−1^.

**Figure A9:**
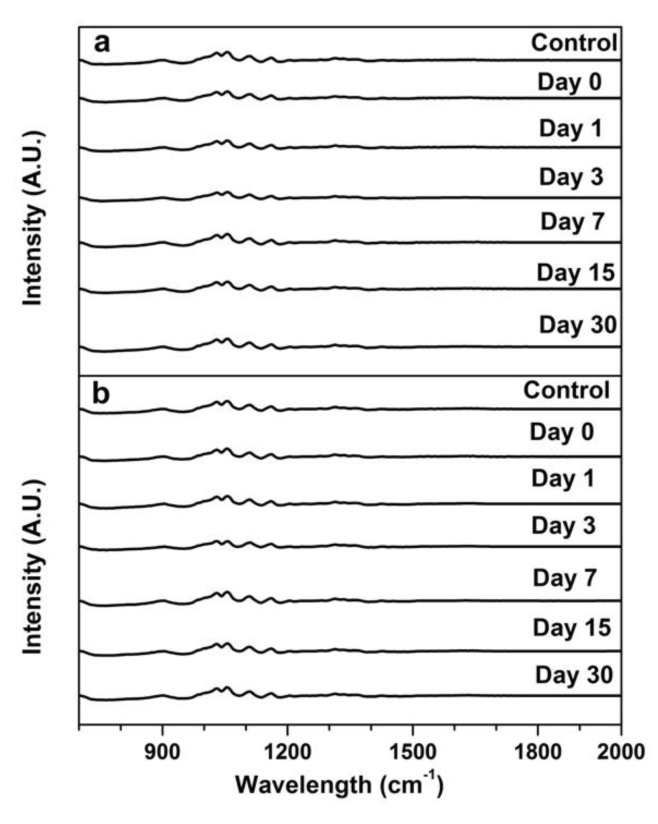
ATR-FTIR spectra of control (**a**) and plasma-treated (**b**) W 3 (cotton) in the range between 700 and 2000 cm^−1^.

In order to determine the chemical change of the wipe samples over storage time in ADBAC (ATR-FTIR spectrum in Figure A6) solution, FTIR spectroscopy measurements were performed with untreated/plasma treated in water/ADBAC immersed wipe samples. The ATR-FTIR spectrum (Figure 2) displays the polyester profile peaks in untreated W1 wipe at about 1712 cm^−1^ assigned to carbonyl (C=O) stretching vibration in ester, at 1234 cm^−1^ appointed to asymmetric stretching of aromatic ester, at 871 cm^−1^ attributed to a carbon-carbon bond (C–C) out of plane bending mode of the benzene rings, and at 720 cm^−1^ associated to the aromatic hydrogen bond (C–H) bending vibrations [34]. W2 and W3 show similarities in the ATR-FTIR spectra. A typical band such as the one at around 2900 cm^−1^ assigned to the cellulose structure’s symmetric stretching vibrations of hydrogen bond (C–H) as well as strong peaks at 1150, 1100 and 1020 cm^−1^ of the glycoside bridges C–O–C bond vibrations and broad bands at 3340 and 3270 cm^−1^ indicating the hydroxyl (O–H) group in stretching vibration mode [35,36,37]. However, in W2 no peak of the polyester appears in the FTIR spectra due to the strong intensity of the cellulose peaks covering the polyester ones. The FTIR result did not exhibit any significant change over storage time or plasma treatment for all the tested wipes (Figure A7, Figure A8 and Figure A9 in the Appendix A), indicating that FTIR is not sensitive enough in detecting the chemical changes after plasma treatment since penetration depth of FTIR is around 2 µm [38]. FTIR-ATR is not able to detect the small amount of ADBAC (Figure A6 in the Appendix A) on the wipes as well as changes in the non-covalent bonds such as ionic, hydrogen, van der Walls or electrostatic forces that can significantly influence the adsorption of the active ingredient. Therefore, the XPS technique was employed to further investigate and quantify the physico-chemical modifications induced by the ADABC adsorption and by the plasma treatment.

### 3.4. Breaking Force and Elongation at Break Over Storage Time

**Figure 3:**
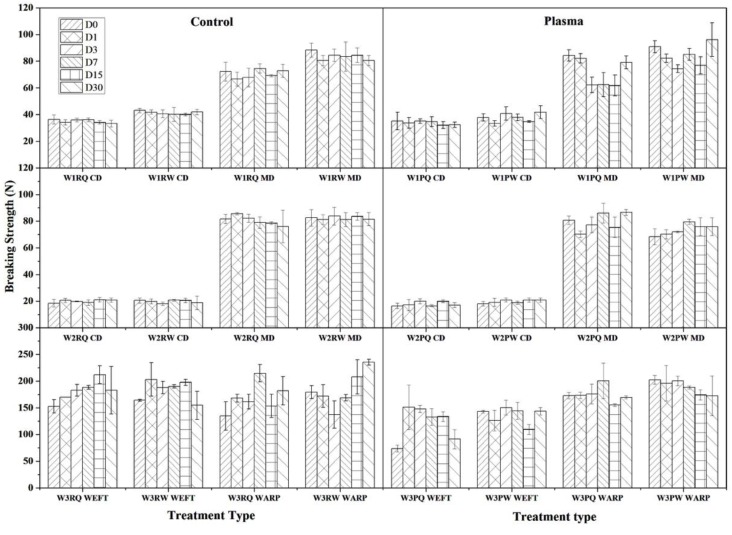
Breaking force (N) change of control (R) and plasma-treated (P) wipe samples during 30 days of storage time. (D0 to 30 represented 30 min, 1, 3, 7, 15, and 30 days’ immersion time).

Wipes used for disinfection purpose should exhibit high tear resistance and tensile strength, low elongation and good abrasion resistance allowing even heavy dirt removal without fiber shedding or breaking and size deformation in the cleaning process. Breaking force and elongation at break of the wipe samples were tested in cross direction (CD) and machine direction (MD) for nonwovens (W1 and W2) and in warp and weft directions for woven structure (W3). As expected, the breaking force is greater in machine direction than in the cross direction in both nonwoven wipe samples (Figure 3). W1 showed double breaking force in CD direction than W2 attributable to the wipes production feature. After plasma treatment, a slight decrease in the breaking force is noted but with no significant changes during ageing. In W3 the ADBAC seems to have a small effect in increasing the force during the time up to 7 days compared to water. Plasma treatment clearly has an effect in weft direction reducing the force at break in water and ADBAC treated wipes of about 26% and 33%, respectively. No significant difference can be noted in the warp direction. This decrease in breaking force can be explained by the reduction in inter-fiber friction after plasma treatment. As observed by LSM, inter-fiber frictional forces of the plasma-treated W3 wipe decreased by the smothering of the cotton fiber surface by etching. Lower forces are needed to overcome the decreased inter-fiber friction resulting in lower breakage loads [39,40].

**Figure 4:**
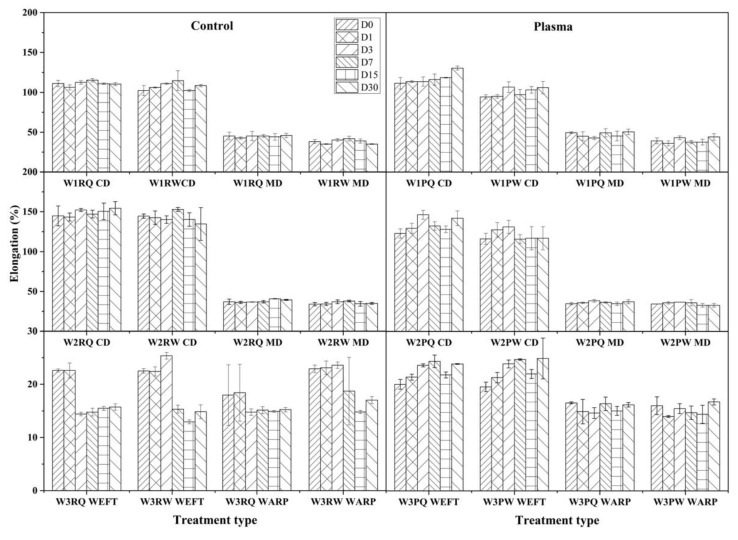
Elongation at break (%) change of control (R) and plasma-treated (P) wipe samples during 30 days of storage time. (D0 to 30 represented 30 min, 1, 3, 7, 15, and 30 days’ immersion time).

Elongation at break is larger in CD than in the MD for all the nonwoven wipes (Figure 4). The plasma-treated wipes showed a slight decrease in CD but not in MD directions. Control W3 shows a clear decrease in elongation in function of the storage time in water after 7 days and in ADBAC after 3 days. The storage of wet wipes clearly has a significant effect on the reduction of the elongation due to the swelling of fibres which restrict the movement of the yarns resulting in a significant loss in elasticity (8%). As previously observed, quaternary ammonium salts, enhancing fibres swelling and accelerate the ageing of the fibres reducing the time in which the fibres lost their elasticity [41]. Surprisingly, plasma-treated W3 in weft direction did not show any loss but a slight increase in elasticity during storage time. This is in accordance with the previously observed decrease in breaking force in the weft direction. No significant changes can be depicted in the warp direction.

### 3.5. Dynamic Mechanical Analysis Over Storage Time (DMA)

**Figure 5:**
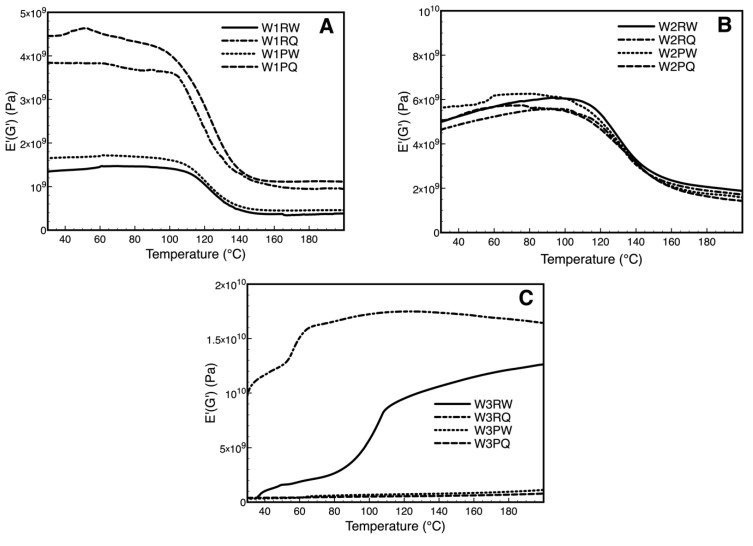
Temperature dependence at 4 Hz of storage (E’) modulus of W1 (**A**), W2 (**B**), W3 (**C**) of untreated (R) and plasma-treated samples (P) at Day 7 immersed in water and ADBAC.

**Figure A10:**
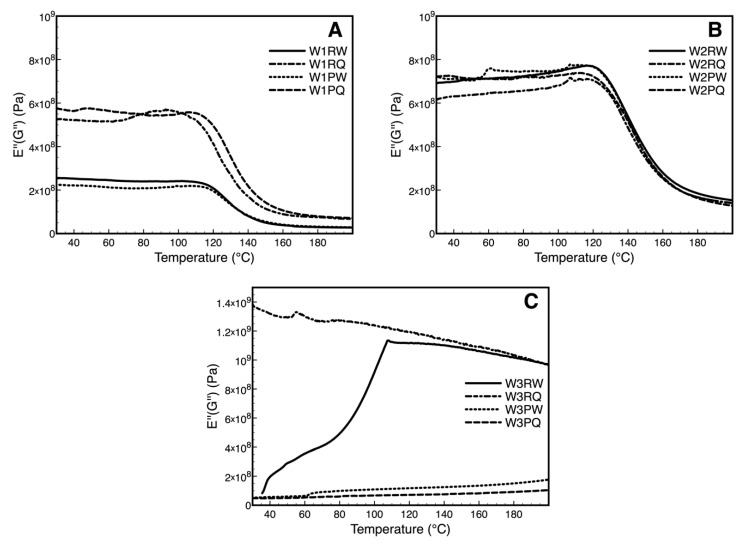
Temperature dependence at 4 Hz of loss (E’’) modulus of W1 (**A**), W2 (**B**), W3 (**C**) of untreated (R) and plasma-treated samples (P) at Day 7 immersion in water and ADBAC.

To study the thermo-mechanical properties of the wiping materials over storage time without and with plasma treatment, the dynamic mechanical analysis (DMA) was performed. Loss modulus, storage modulus and tan delta are the DMA parameters, providing profile information regarding the viscoelastic properties of the polymer, relaxation process, structural hetero groups, molecular motion, and morphology of the polymer blends [42]. Storage modulus (E’) describes the stored energy in the polymer, which reflects the measure of elastic response of a material, while loss modulus (E’’) defines the energy dissipated as heat, representing the plastic response. Figure 5 display the storage modulus of control wipe samples (R) and their plasma-treated wipe samples (P) at Day 7. W1 (100% polyester) control and plasma-treated samples show a significant difference for water and ADBAC immersion treatment. In the untreated wipe sample, the storage modulus at 30 °C increased three times with the addition of ADBAC from 1.3 GPa to 3.8 GPa. Meanwhile, in the plasma-treated wipe sample, the storage modulus increased from 1.7 GPa to 4.5 GPa. The increased storage energy in ADBAC immersed wipe samples shows that the quaternary ammonium salt is able to alter the intermolecular bonding that hinders the mobility of polymer chains in the wipes [43]. This value maintained relatively stable up to 100 °C. After this temperature, all samples storage modulus starts to decrease due to the increased mobility of the polymer chains. However, ADBAC samples (untreated and plasma-treated) display a higher decrease to values similar to water storage modulus probably due to the rapid ADBAC molecule degradation. Plasma-treated wipe samples give a higher storage modulus than the control wipe in either ADBAC or water samples. The improvements in the thermo-mechanical properties of the plasma-treated wipes can be associated to the improved adhesion among fibers in the interface region promoted by the plasma generated species such as free radicals and other oxidized functional groups introduced on the surface of the polyester wipes [44]. W2 wipes (Figure 5B) did not show any significant differences among all the sample behaving like a composite blend showing both the mechanical properties of cellulose and polyester at the same time. Untreated W3 wipes display significant differences between ADBAC and water immersed samples due to the interaction between ADBAC and cellulose. The ADBAC immersed wipe sample shows higher storage energy than the one immersed in water resulting from the electrostatic forces between the quaternary ammonium salt and the cellulose structure [45,46]. Despite the XPS analysis did not show significant oxidation of the cellulose surface, plasma-treated W3 wipes showed dramatic changes in the thermo-mechanical properties in both water and ADBAC immersed wipes (Figure 5C). Contrarily to W1, the control W3 wipe exhibits a much higher storage modulus than the plasma-treated one (1 GPa vs. 0.3 GPa). This change may result from the etching effect of plasma treatment on cotton fabric. The etching effect smoothens the cotton fabric surface by removing crosslinked impurities and surface cellulose microfibrils, as previously discussed, leading to a decrease in storage modulus. Similar behavior was observed for all the wipes in the loss modulus (Figure A10 in Appendix A).

**Figure 6:**
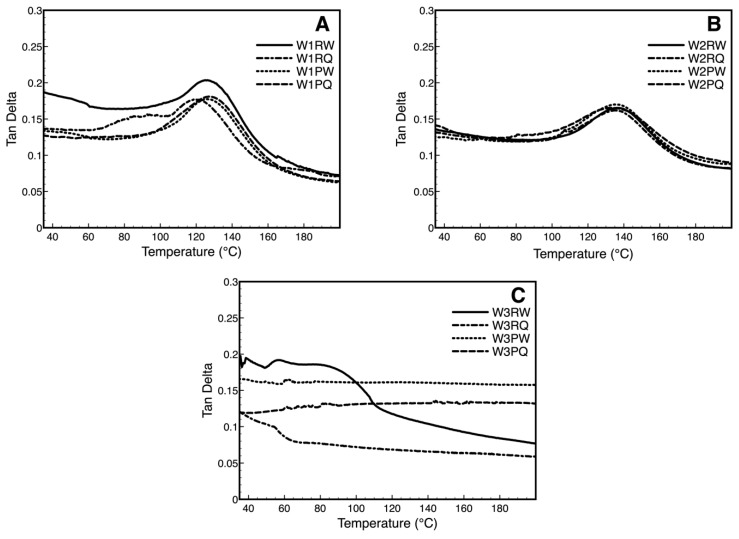
Temperature dependence at 4 Hz of tan delta of W1 (**A**), W2 (**B**), W3 (**C**) of untreated (R) and plasma-treated samples (P) at Day 7 immersed in water and ADBAC.

The tan delta, also known as the damping factor is the ratio between the loss (E’’) and storage modulus (E’) and it is an indicator of the molecular motions in a material. A low tan δ value exhibits a material possessing a more elastic strain component, on the contrary, a high value implies a more non-elastic feature. The presence of ADBAC and plasma treatment results in a decrease in tan δ in W1 samples (Figure 6A) due to the stress transfers between wipe and the ADBAC and the plasma introduced functional groups altering the intermolecular bonding that change the mobility of polymer chains in the wipe. The decrease in the magnitude of tan δ upon addition of ADBAC or plasma treatment to the wipes suggests limited mobility of polymeric chains of polyester because of the interactions of the ADBAC or by the plasma-generated functional groups that cause the decrease in damping factor [47]. The glass transition temperature shift of the ADBAC in untreated control to lower temperature confirm the plasticizing effect of the quaternary ammoniums salt on the polymeric network only when no plasma-generated oxygen species are present [48,49]. As expected, W2 did not show a significant difference in tan δ values with different treatments suggesting that the overall viscoelastic properties of the blend are not perturbed by the ADBAC immersion and plasma treatment probably due to the absorption/adsorption ability of cellulose compensating the interaction of the ADBAC and plasma-generated species in polyester. W3 wipe is clearly the most affected in its thermo-mechanical properties by ADBAC immersion and plasma treatment. Control W3 immersed in water displays a decrease in tan δ values indicating a more elastic property by the rise of temperature. However, the ADBAC immersed control samples gives a much lower tan δ value due to the interaction between ADBAC and cellulose. It seems that the electrostatic forces interactions enhance the elastic property of cellulose. However, the influence from the interaction is getting weak by raise of the temperature, especially after 110 °C. Surprisingly, the tan δ values of plasma-treated W3 samples were found to be temperature independent both for water and ADBAC samples. The plasma effect in cotton clearly increases the non-elastic strain component due to the etching effect that removes the crosslinked impurities and the entangled micro-fibrillated structures as previously discussed.

### 3.6. Antimicrbial Efficacy

**Figure 7:**
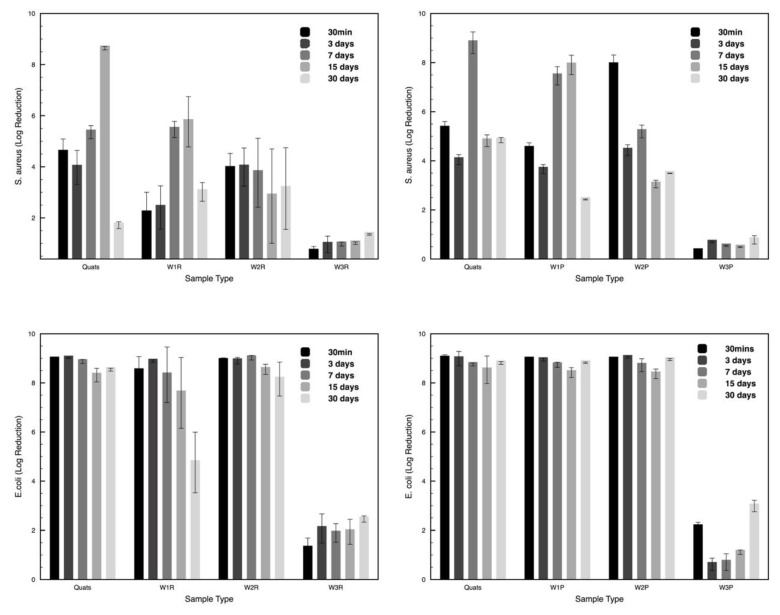
Log reduction of *S. aureus* and *E. coli* on the untreated (R) and plasma-treated (P) disinfecting wipes stored for 30 min, 3, 7, 15, and 30 days.

The antimicrobial efficacy (ASTM E2149-13a) as Log reductions are presented in Figure 7 in the function of the storage time (30 min–30 days) and bacterial strain (Gram-positive and Gram-negative). This test method allows direct and complete contact between microorganism and active ingredients, which eliminates interferences from other parameters (i.e. mechanical action or surface contact only). The chosen method gives a straightforward observation of how the interaction impacts on the biocidal effect. ANOVA analysis shows that storage time does not have any significant influence on the antimicrobial efficacy of the control and plasma-treated wipe samples (Table A1 and Table A2 in Appendix A). However, sample type has a significant influence on the Log reduction (control wipe with *p*-value 6.30615 × 10^−5^ and plasma-treated wipe sample with *p*-value 2.55926 × 10^−5^). Due to the adsorption of ADBAC active ingredient on cellulose material, untreated and plasma-treated W3 showed the lowest Log reduction among all the testing samples. The Log reduction result corresponds to the previously observed quaternary ammonium salt inactivation in cotton due to the adsorption on the fibers that not release the antimicrobial agents [8,50]. The adsorbed active ingredients lost its bactericidal function (Log reduction <3) and thereby failed the antimicrobial efficacy test. In the case of the other W1 and W2, the wipes are acting like a carrier that transfers the ADBAC solution from the bulk onto the target surface. Thus, the Log reduction shows almost the same result as the pure ADBAC solution. In addition, ADBAC is clearly more effective in Gram-negative *E. coli* than gram-positive *S. aureus* for every type of wipe including plasma-treated ones. ANOVA analysis of untreated W1 and W2 shows that the storage time has a significant influence on their Log reduction of *E. coli* (control wipe samples with a *p*-value of 0.036 and plasma-treated wipe samples with a *p*-value of 0.005).

Despite the great variability of the antimicrobial effect, plasma treatment displays a significant effect in W1 and W2 wipes for *S. aureus* and for W1 for *E. coli*. In *S. aureus* plasma-treated W1 wipe enhance the antimicrobial efficacy increasing the Log reduction from 2.5 to 4.5 at day 1 and from 6 to 8 after 15 days while after 30 days both untreated and plasma-treated samples show a significant reduction in antimicrobial efficacy. Similar behaviour can be observed in W2 wipe but only up to 7 days. In the case of *E. coli,* the plasma treatment seems to preserve the antimicrobial activity during storage time for W1 wipe while no changes in W2 can be depicted. As expected, plasma treatment did not affect the very low antimicrobial activity in cotton-based W3 wipe. Plasma treatment in polyester wipes (W1) is able to minimize the main drawback on ADBAC absorption, namely the hydrophobicity of the polyester surface which does not allow ADBAC to remain on the wipe. Plasma treatment improve the surface adsorption of ADBAC due to the plasma-generated oxygen species that allow a controlled release of the disinfectant over storage time conditions [51].

## 4. Conclusions

XPS analysis demonstrates the presence of relevant plasma-generated oxygen species on W1 and W2 wipes while LSM demonstrates the plasma etching effect in smoothing the cotton surface of W3 wipe. Plasma treatment also shows to have a significant effect on the thermos-mechanical properties of the wipes slightly reducing the force at break and elongation in water and ADBAC treated wipes. Plasma-treated W3 cotton wipe did not show any loss but a slight increase in elasticity during storage time in weft direction due to the reducing of the cellulose fiber swelling. DMA analysis demonstrates that the blend wipe (W2) is not affected in its viscoelastic properties and highlights the opposite mechanical behavior of W1 and W3 wipes. The presence of plasma treatment in W1 improve the elastic response of the wipe limiting the mobility of the polymeric chains of polyester, while the plasma treatment in W3 clearly increases the non-elastic strain component due to the etching effect. DBD plasma treatment is able to duplicate the shelf life in term of antimicrobial efficacy of pure polyester wipes (W1) up to 15 days for Gram-positive bacteria and 30 days for Gram-negative bacteria compared to the untreated samples. In a less extend also in the blend polyester /cotton wipe (W2) plasma treatment is able to enhance the antimicrobial efficacy of about 30% for Gram-positive bacteria and continue to have excellent activity in Gram-negative bacteria. The adsorption of ADBAC on cellulose (W3) completely block the biocidal effect of active ingredients, which is a high risk for infection control.

## Figures and Tables

**Table 1:** Information of material, structure, dimension, fabric thickness and areal density. The data was reported as average of 10/5 repetitions including their coefficients of variance in percentage (CV%).

Sample	Components	Structure	Dimension (cm) of a 0.05 g Sample	Fabric Thickness (mm ± CV%)	Areal Density (g m^−2^ ± CV%)
**W1**	100% polyester	Hydroentangled nonwoven	2.5 × 4.5	0.36 ± 5.93	40.92 ± 1.59
**W2**	55% cellulose 45% polyester	Hydroentangled nonwoven	2.5 × 2.5	0.54 ± 3.02	69.58 ± 0.96
**W3**	100% bleached cotton	1/1 plain weave	2.5 × 1.5	0.98 ± 5.09	118.72 ± 0.41

**Table 2:** Relative chemical composition and atomic ratio of untreated and dielectric barrier discharge (DBD) plasma-treated wipes.

	Untreated					Plasma Treated				
	Chemical Composition (%)			Atomic Ratio		Chemical Composition (%)			Atomic Ratio	
	C	O	N	O/C	N/C	C	O	N	O/C	N/C
W1	73.3	26.2	0.5	0.36	0.01	65.2	33.2	0.7	0.51	0.01
W2	69.1	30.5	0.4	0.44	0.01	65.2	33.8	1.1	0.52	0.02
W3	61.9	37.7	0.4	0.61	0.01	60.7	38.7	0.6	0.64	0.01
W1Q	82.2	15.1	1.7	0.18	0.02	79.2	18.5	2.2	0.23	0.03
W2Q	60.8	38.6	0.6	0.63	0.01	61.3	38.2	0.5	0.62	0.01
W3Q	62.9	36.1	1.0	0.57	0.02	62.3	37.0	0.7	0.59	0.01

## Appendix A

**Table A1:** ANOVA analysis of antimicrobial test result over storage time: control wipe samples.

Source of Variation		SS	df	MS	F	*P*-value	F crit
**ANOVA result of *S. aureus***	Sample Type	54.160191	3	18.053397	19.676001	6.30615 × 10^−5^	3.490295
	Storage Time	6.8844590	4	1.7211148	1.8758052	0.179495939	3.259167
	Error	11.010406	12	0.9175339			
	Total	72.055057	19				
**ANOVA result of *E. coli***	Sample Type	149.61623	3	49.872078	68.638708	7.98187 × 10^−8^	3.490295
	Storage Time	2.3886352	4	0.5971588	0.8218669	0.535746027	3.259167
	Error	8.7190589	12	0.7265882			
	Total	160.723929	19				

**Table A2:** ANOVA analysis of antimicrobial test result over storage time: plasma treated wipe samples.

Source of Variation		SS	df	MS	F	P-value	F crit
**ANOVA result of *S. aureus***	Sample Type	74.189835	3	24.729945	23.570227	2.55926 × 10^−5^	3.4902948
	Storage Time	18.446129	4	4.6115324	4.3952731	0.02034882	3.2591667
	Error	12.590432	12	1.0492027			
	Total	105.22640	19				
**ANOVA result of *E. coli***	Sample Type	156.03905	3	52.013016	54,090543	3.03227 × 10^−7^	3.4902948
	Storage Time	22.149016	4	5.5372540	5.7584253	0.00798589	3.2591667
	Error	11.539100	12	0.9615917			
	Total	189.72717	19				
